# Attachment Style and Self-Experience: The Association Between Attachment Style and Self-Reported Altered Self-Experience in Patients With Psychotic Disorders, Unaffected Siblings, and Healthy Controls

**DOI:** 10.1097/NMD.0000000000001634

**Published:** 2023-03-24

**Authors:** Justine de With, Lieuwe de Haan, Frederike Schirmbeck

**Affiliations:** ∗ Early Psychosis Department, Amsterdam UMC (location AMC), Department of Psychiatry; † Arkin Institute for Mental Health, Amsterdam, the Netherlands.

**Keywords:** Disturbed self-awareness, depersonalization, schizophrenia spectrum disorders

## Abstract

The present study aimed to examine the cross-sectional association between attachment style and self-reported disturbed self-awareness (disturbed sense of mineness of experiences) and depersonalization (disturbed sense of first-person perspective) in patients with psychotic disorders, unaffected siblings, and healthy controls. Data pertain to a subsample of the GROUP (Genetic Risk and Outcome of Psychosis) study. We found positive associations between anxious attachment and disturbed self-awareness and depersonalization across participants with different psychosis vulnerability. We also found a positive association between avoidant attachment and depersonalization, although on a trend level. Findings indicate that attachment style is associated with self-reported disturbed self-awareness and depersonalization over and above the influence of psychotic or depressive experiences in people across the vulnerability spectrum of psychosis. This supports the importance of attachment style, self-awareness, and depersonalization as potential targets in prevention and treatment interventions in patients with psychotic disorders or those with increased vulnerability.

Altered self-experiences are seen as a fundamental feature of schizophrenia spectrum disorders (Sass et al., 2013; Sass and Parnas, 2003) and may underlie several symptom dimensions such as positive, negative, and disorganized psychotic symptoms (Parnas et al., 2005). Altered self-experiences capture a disturbed sense of self-presence and first-person perspective, disturbed self-awareness, and disturbed common sense, among other experiences (Sass et al., 2013; Sass and Parnas, 2003). Several studies, including a recent systematic review (Henriksen et al., 2021) and three meta-analyses (Burgin et al., 2022; Hur et al., 2014; Raballo et al., 2021), consistently show that altered self-experiences are more common in schizophrenia spectrum disorders (including schizophrenia, nonaffective psychotic disorders, and schizotypal disorders) compared with other mental disorders (Henriksen et al., 2021; Raballo et al., 2021) or healthy controls (Burgin et al., 2022; Henriksen et al., 2021; Hur et al., 2014; Raballo et al., 2021).

In the present study, we are interested in subaspects of altered self-experiences and more specifically in disturbed self-awareness and depersonalization. Disturbed self-awareness refers to the implicit awareness of an individual of being the owner of his body, perceptions, thoughts, and experiences (sense of ownership), as well as an individual experiencing himself as the source or initiator of his thoughts, intentions, and actions (sense of agency) (Zandersen and Parnas, 2019). Disturbed self-awareness in schizophrenia spectrum disorders is reflected in a reduced sense of ownership and agency and is hypothesized to have three main aspects: diminished self-affection, hyperreflexivity, and disturbed grip or hold (Sass, 2014; Sass and Parnas, 2003). Diminished self-affection refers to a decline in the experienced sense of existing as a subject of awareness or a decline in sense of ownership or agency. Hyperreflexivity refers to an intensified awareness of phenomena that are normally in the background of experience or considered as part of oneself. Both self-awareness disturbances may lead to a disturbed sense of grip or hold on the inside and outside world (Parnas et al., 2005). Depersonalization refers to a feeling of detachment from one's self (Parnas et al., 2005). In previous studies, depersonalization is often considered in the light of dissociative phenomena (Berry et al., 2017; Laoide et al., 2018; Mertens et al., 2021; Sandberg, 2010; Sheinbaum et al., 2014; Sheinbaum et al., 2020; van Dam et al., 2014); however, in the current study, we are interested in depersonalization in the form of a disturbed sense of self-presence and first-person perspective (Parnas et al., 2005).

Recent studies consider altered self-experiences as important factors in the development of psychotic disorders (Cicero et al., 2017; Gawęda et al., 2018), hence knowledge concerning possible etiological factors associated with altered self-experiences is important. There is a strong rationale to hypothesize an interrelation between attachment style and altered self-experiences. Experiences in (early) social interactions may affect how we see ourselves, others, and the world. The deeply ingrained beliefs or perceptions individuals hold about themselves and others are embedded in attachment theory (Bowlby, 1969). Attachment theory proposes that the pattern of interaction between infants and their primary caregivers leads to the development of mental representations about self and others (Ainsworth, 1978). These representations function as templates for thoughts, feelings, and behaviors in interactions with others throughout the lifespan (Brennan et al., 1998). Securely attached individuals have a secure foundation and believe that attachment figures are available when needed. They possess the ability to establish stable and long-term intimate relationships (Brennan et al., 1998). An insecure attachment style has been conceptualized in two underlying dimensions: anxious attachment and avoidant attachment (Brennan et al., 1998). Anxious attachment is characterized by a continuous desire for proximity and fear of rejection, making physical or emotional distance difficult to tolerate. Avoidant attachment is characterized by a continuous desire for independence and a fear of proximity, making physical or emotional closeness difficult to tolerate (Brennan et al., 1998). Previously, Gawęda et al. (2018) found both insecure attachment and self-disturbances to play a mediating role in the association between trauma and development of psychotic symptoms in a nonclinical population. The authors also reported a significant interrelation between anxious attachment and self-disturbance (Gawęda et al., 2018). Moreover, previous studies found positive associations between insecure attachment and dissociative phenomena (Laoide et al., 2018; Sandberg, 2010).

However, we are not aware of a study that explicitly investigated the association between attachment style and self-reported self-awareness or depersonalization in patients with psychotic disorders. Hence, in the present study, we aimed to examine this association in people with different vulnerability for psychosis, that is, in a combined sample of patients with a nonaffective psychotic disorder, unaffected siblings, and healthy controls. We hypothesized that a more insecure attachment style is associated with higher levels of disturbed self-awareness and depersonalization, and accounts for unique variance in these disturbances over and above the influence of psychotic and depressive experiences.

Ultimately, enhancing our understanding of factors associated with disturbed self-awareness or depersonalization may be informative for prevention and treatment interventions for persons with, or at risk of, psychotic disorders. Being aware of insecure attachment and its possible relation to the development of altered self-experiences may enlarge our empathic understanding of the experiential world of patients. This might strengthen the focus on the therapist-patient relationship and improve care with a positive effect on the development of a coherent and stable sense of self and a reduction of altered self-experiences.

## METHODS

### Study Design and Participants

Patients included in the current study were part of the Genetic Risk and Outcome of Psychosis (GROUP) study. GROUP is a Dutch multisite naturalistic follow-up study (after 3 and 6 years) designed to study risk and protective factors influencing the onset and course of psychotic disorders in patients, their unaffected family members, and nonrelated controls (Korver et al., 2012). Patients were recruited in selected representative geographical areas of the Netherlands and (the Dutch-speaking part of) Belgium. Random mailings to addresses were used to recruit controls in the selected areas. Interviewers received extensive training to optimize the reliability of measurements (Korver et al., 2012). Inclusion criteria were an age range from 16 to 50 years and mastery of the Dutch language. Patients had to meet the *DSM-IV-TR* criteria for a nonaffective psychotic disorder, assessed with the Comprehensive Assessment of Symptoms and History (Andreasen et al., 1992) or the Schedules for Clinical Assessment in Neuropsychiatry (Schutzwohl et al., 2007). The assessments were conducted by trained researchers. Participants were either investigated on site or (if necessary) assessments were conducted at their home. The same protocol was being used during in-home assessments and assessments on site. Average assessment time was 4 hours for patients and 3 hours for siblings and controls. The self-reported questionnaires (including the Psychosis Attachment Measure [PAM] and Self-Experience Lifetime Frequency Scale [SELF]) were sent to the participants before the assessment meeting in order for them to fill out and bring the questionnaires to the meeting. At the start of the assessment, the self-report questionnaires were checked for missing data. The full procedure of the study has been described elsewhere (Korver et al., 2012). The current add-on study had a cross-sectional design, using data from the second follow-up assessment (after 6 years). Participants from the Amsterdam site of GROUP participated in this study concerning attachment style, disturbed self-awareness, and depersonalization. We included 74 patients, 78 siblings, and 32 controls; thus the combined sample was 184 participants. Sociodemographic characteristics at baseline are presented in Table 1. The sample consisted of 112 males and 70 females with a mean age of 35.6 years (ranging from 22 to 61 years). Most participants identified as White, whereas others were Moroccan, Surinamese, Turkish, of another ethnicity, or mixed.

**TABLE 1:** Baseline Characteristics of the Total Sample and Separate Groups (Patients, Siblings, and Controls)

	Total Sample (*n* = 184)	Patients (*n* = 74)	Siblings (*n* = 78)	Controls (*n* = 32)
Age in years	35.59 (9.04)	34.85 (8.05)	35.24 (8.53)	38.13 (11.86)
Gender, n				
Male	112	62	33	17
Female	70	12	44	14
Unknown	2	0	1	1
Ethnicity, n				
White	153	59	66	28
Non-white	26	12	11	3
Unknown	5	3	1	1
Education in years	17.00 (3.60)	16.85 (4.23)	17.04 (3.03)	17.23 (3.42)
Cannabis use, n				
Yes	14	5	6	3
No	143	57	61	25
Unknown	27	12	11	4
CAPE				
Positive symptoms	0.23 (0.34)	0.43 (0.44)	0.09 (0.12)	0.08 (0.12)
Negative symptoms	0.61 (0.47)	0.87 (0.52)	0.45 (0.37)	0.39 (0.28)
Depressive symptoms	0.62 (0.49)	0.87 (0.53)	0.49 (0.40)	0.38 (0.32)
*DSM-IV* diagnosis, n				
Schizophrenia		37		
Schizophreniform disorder		1		
Schizoaffective disorder		15		
Psychotic disorder		13		
Delusional		1		
Other		8		
Illness duration in years		11.18 (3.85)		
Antipsychotic medication				
Yes		48		
No		2		
Unknown		24		

Data are in mean (SD) unless stated otherwise.

### Measurements

#### Disturbed Self-Awareness and Depersonalization

The SELF was used to assess self-reported self-experience phenomena (Heering et al., 2016) (see Supplementary Table S1, Supplemental Digital Content 1, http://links.lww.com/JNMD/A159). The SELF captures two aspects of altered self-experience, namely, disturbed self-awareness and depersonalization. Eight items represent the domain disturbed self-awareness, and six items represent the domain depersonalization. The SELF domain disturbed self-awareness contains items based on the Examination of Anomalous Self-Experience (EASE) (Henriksen et al., 2021) and the Comprehensive Assessment of At-Risk Mental States (Yung et al., 2005). The SELF domain depersonalization contains items from the Depersonalization Severity Scale (Simeon et al., 2001). In the EASE, comparable items are captured under items 2.2 “distorted first-person perspective” and 2.3 “other states of depersonalization” (Henriksen et al., 2021). Patients were asked to report about the life-time frequency and level of distress of the 12 items on a 5-point Likert rating scale. Frequency scores range from 0 (never) to 4 (continuously) and level of distress scores from 1 (no distress) to 5 (extremely distressed). A total score of the two subscales was calculated by adding the scores of both frequency and distress for each item, with higher scores reflecting higher levels of disturbed self-awareness and depersonalization. Good internal consistency of the two components has previously been demonstrated in a combined clinical and nonclinical sample, with Cronbach's α of the two SELF domains ranging from 0.79 to 0.88 (Heering et al., 2016).

### Attachment Style

Attachment style was measured with the Dutch version of the PAM (Korver, 2014). The Dutch PAM is a 15-item self-report questionnaire with seven items reflecting avoidant attachment and eight reflecting anxious attachment. Items are rated on a 4-point Likert rating scale ranging from 0 (not at all) to 3 (very much) (Korver, 2014). Average item scores were calculated for avoidant and anxious attachment, with higher scores reflecting higher levels of insecure attachment (Berry et al., 2006; Korver, 2014). The Dutch PAM is observed to be an adequate measurement with high construct validity and reliability in a clinical and nonclinical sample. Cronbach's α of the two PAM domains ranged from 0.70 to 0.83 (Korver, 2014).

### Covariates

Sociodemographic data were evaluated using a self-reported questionnaire, specifically developed for the GROUP study. To correct for possible confounding effects, covariates were selected a priori. Age and gender were added as covariates, as well as positive symptoms, negative symptoms, and depressive symptoms, based on their putative association with attachment style or self-disturbance (Carr et al., 2018; Gumley et al., 2014; Haug et al., 2015; Pearse et al., 2020; Rasmussen et al., 2020; Værnes et al., 2022; van Dam et al., 2014). The frequency of psychotic experiences was measured with the Community Assessment of Psychotic Experiences (CAPE) (Konings et al., 2006). This self-report questionnaire assesses the frequency of psychotic and depressive symptoms over the past 3 years. A mean total score was calculated for the subscales positive symptoms, negative symptoms, and depressive symptoms, with a higher score reflecting a higher frequency of symptoms. According to a meta-analysis, Cronbach's α values of the three CAPE domains reported in previous studies had a meta-analytic mean ranging between 0.81 and 0.91 (Mark and Toulopoulou, 2016).

### Statistical Analyses

All analyses were performed using Statistical Package for the Social Sciences (IBM SPSS Statistics) version 28.0. A power analysis (desired probability level of 0.025 and statistical power level of 0.8, 6 predictors per model and an anticipated effect size of 0.15) was performed to estimate the necessary sample size. The anticipated effect size is determined based on previously found intercorrelations by Gawęda et al. (2018). The estimated minimum required sample size was 114. As our combined sample consisted of 184 participants, we assume our regression analyses have sufficient power to detect significant findings. A missing value analysis was conducted for item scores on the PAM and SELF (see Supplementary Table S2, Supplemental Digital Content 1, http://links.lww.com/JNMD/A159). To generate subscale scores for all participants, missing items were imputed with the logistic regression method using the Markov chain Monte Carlo algorithm. The number of imputations was set at five based on the low percentage of incomplete items. Outliers were not removed because we expected scores to vary greatly in the field, which should be reflected in our data. To check the assumptions of normality, linearity, and homoscedasticity, we used probability plots and scatterplots of standardized residuals. Violations of normality were detected for both attachment style as well as disturbed self-awareness and depersonalization data, with positively skewed distributions, indicating clustering of scores at the lower ends. We compared the results of hierarchical linear multiple regression analyses after log transformation and square root transformation of the dependent and independent variables. Since the results of the untransformed and (log and square root) transformed models were similar, we chose to report only results of the models without transformation of the variables. Sample characteristics of participants were assessed using descriptive statistics. Correlations between attachment style and altered self-experience domains of the total sample and separate groups (patients, siblings and controls) were assessed using Pearson correlation (Supplementary Table S3, Supplemental Digital Content 1, http://links.lww.com/JNMD/A159).

First, between-group differences in the association between attachment style and self-reported disturbed self-awareness and depersonalization were tested by using linear regression models. Therefore, attachment style × status group interaction effects were tested for the two outcome domains. Because these models did not show significant differences between status groups (see Table 2), we subsequently fitted models investigating the association between attachment style and disturbed self-awareness and depersonalization for the combined sample of participants.

**TABLE 2:** Results of Multiple Linear Regression Models Regarding the Interaction Between Group Status and Attachment Style and Self-Reported Disturbed Self-Awareness and Depersonalization

	SELF Disturbed Self-Awareness		SELF Depersonalization	
	β (SE)	*p*	β (SE)	*p*
PAM anxiety				
Constant	−1.533 (1.997)	0.444	−0.205 (1.300)	0.875
Group status	5.026 (1.501)	0.001*	1.893 (0.977)	0.054
PAM anxiety	3.155 (3.459)	0.363	3.237 (2.251)	0.152
Group status × PAM anxiety	2.293 (2.193)	0.297	1.248 (1.427)	0.383
PAM avoidance				
Constant	−2.900 (3.062)	0.345	−0.599 (1.983)	0.763
Group status	4.812 (2.303)	0.038	0.964 (1.491)	0.519
PAM avoidance	2.868 (3.159)	0.365	1.916 (2.046)	0.350
Group status × PAM avoidance	2.016 (2.193)	0.359	2.036 (1.420)	0.153

*Significant after Bonferroni correction (*p* < 0.025).

Second, hierarchical linear multiple regression analyses were carried out to investigate the association between attachment style and disturbed self-awareness and depersonalization, with attachment styles as predictors and disturbed self-awareness and depersonalization as outcome measures. The a priori selected covariates were first added to the model in steps, examining whether they contributed to the model, while controlling for covariates in previous steps. In the first model, age and gender were entered. In the second model, we entered the other covariates in the aforementioned order before adding attachment style at the last step. All analyses were performed with separate models for anxious and avoidant attachment. Multicollinearity was assessed by calculating the variance inflation factor for all models. To account for multiple testing, we used a Bonferroni correction to minimize the risk of type I errors. Therefore, the two-tailed significance threshold was set at 0.025 (0.05 divided by 2, given the two SELF outcome domains).

## RESULTS

### Clinical Characteristics

Clinical characteristics regarding attachment style and self-reported disturbed self-awareness and depersonalization are presented in Table 3. Correlations between attachment style and altered self-experience domains of the total sample and separate groups (patients, siblings, and controls) are shown in Supplementary Table S3 (Supplemental Digital Content 1, http://links.lww.com/JNMD/A159). Our results indicate that anxious attachment was more strongly correlated to altered self-experiences than avoidant attachment across groups.

**TABLE 3:** Clinical Characteristics Regarding Attachment Style and Self-Reported Disturbed Self-Awareness and Depersonalization of the Total Sample and Separate Groups (Patients, Siblings, and Controls)

	Total Sample (*n* = 184)	Patients (*n* = 74)	Siblings (*n* = 78)	Controls (*n* = 32)
PAM				
Anxiety	0.57 (0.47)	0.75 (0.51)	0.46 (0.43)	0.40 (0.35)
Avoidance	0.95 (0.47)	1.09 (0.45)	0.87 (0.48)	0.85 (0.40)
SELF				
Disturbed self-awareness	8.25 (4.94)	15.96 (12.24)	2.88 (5.80)	3.50 (5.53)
Depersonalization	4.94 (6.79)	8.85 (7.71)	1.91 (4.12)	3.31 (5.30)

Data are in mean (SD).

### Cross-Sectional Associations Between Attachment Style and Self-Reported Altered Self-Experiences

Cross-sectional associations between attachment style and disturbed self-awareness and depersonalization in the total sample of participants were assessed by using hierarchical linear multiple regression models, while controlling for age, gender, positive symptoms, negative symptoms, and depressive symptoms. The covariates were added to the model in steps; anxious and avoidant attachments were added in the last step. Only the final model is presented here (see Table 4), for the detailed models with all subsequent steps see Supplementary Table S4 (Supplemental Digital Content 1, http://links.lww.com/JNMD/A159). We found that anxious attachment was positively associated with disturbed self-awareness (β = 4.048, *p* = 0.023) and depersonalization (β = 2.975, *p* = 0.011). Positive symptoms were positively associated with disturbed self-awareness (β = 11.899, *p* < 0.001) and depersonalization (β = 4.659, *p* = 0.004). The other covariates (age, gender, negative symptoms, and depressive symptoms) were not significantly associated after applying Bonferroni correction. Avoidant attachment was positively associated with depersonalization on a trend level (β = 2.129, *p* = 0.051). Depersonalization was positively associated with positive symptoms (β = 4.745, *p* = 0.004). Avoidant attachment was not significantly associated with disturbed self-awareness, although avoidant attachment was positively associated with positive symptoms (β = 11.950, *p* < 0.001) and depressive symptoms (β = 5.775, *p* = 0.020).

**TABLE 4:** Results of Hierarchical Multiple Linear Regression Models Regarding the Cross-Sectional Association Between Attachment Style and Self-Reported Disturbed Self-Awareness and Depersonalization of the Total Sample of Participants

	SELF Disturbed Self-Awareness		SELF Depersonalization	
	β (SE)	*p*	β (SE)	*p*
PAM anxiety				
Constant	2.562 (3.195)	0.424	1.476 (2.061)	0.475
Age	0.056 (0.076)	0.461	−0.021 (0.049)	0.667
Gender	−2.916 (1.428)	0.043	−0.620 (0.921)	0.502
CAPE positive symptoms	11.899 (2.492)	<0.001*	4.659 (1.608)	0.004*
CAPE negative symptoms	0.508 (2.647)	0.848	3.765 (1.708)	0.029
CAPE depressive symptoms	3.985 (2.591)	0.126	0.080 (1.671)	0.962
PAM anxiety	4.048 (1.783)	0.023*	2.975 (1.150)	0.011*
PAM avoidance				
Constant	2.088 (3.367)	0.536	0.945 (2.171)	0.664
Age	0.031 (0.075)	0.677	−0.038 (0.049)	0.430
Gender	−2.557 (1.429)	0.075	−0.364 (0.921)	0.693
CAPE positive symptoms	11.950 (2.524)	<0.001*	4.745 (1.628)	0.004*
CAPE negative symptoms	−0.125 (2.802)	0.964	3.151 (1.807)	0.083
CAPE depressive symptoms	5.775 (2.464)	0.020*	1.361 (1.589)	0.393
PAM avoidance	2.537 (1.682)	0.133	2.129 (1.084)	0.051

*Significant after Bonferroni correction (*p* < 0.025).

## DISCUSSION

### Summary of Findings

The current study investigated the cross-sectional associations between attachment style and self-reported disturbed self-awareness and depersonalization in a sample of patients with a nonaffective psychotic disorder, unaffected siblings, and healthy controls. Our main finding is that attachment style is associated with self-reported disturbed self-awareness and depersonalization over and above the influence of psychotic and depressive experiences in people with different vulnerability for psychosis. More specifically, our results show that anxious attachment is associated with self-reported disturbed self-awareness and depersonalization, and avoidant attachment is associated with self-reported depersonalization on a trend level.

### Our Findings Compared With Findings of Previous Research

To the best of our knowledge, this is the first study investigating the association between attachment style and self-reported disturbed self-awareness and depersonalization in people across the psychosis vulnerability spectrum. However, our results build on previous research by Gawęda et al. (2018), who reported a significant association between anxious attachment (measured with the PAM) and self-disorders (measured with the Inventory of Psychotic-like Anomalous Self-experiences) in a nonclinical population. The current study adds to the latter by including both a clinical and nonclinical population, while correcting for age, gender, and positive, negative, and depressive symptoms. Correcting for psychotic symptoms rather than using psychotic symptoms as outcome measures allowed us to study associations between attachment style and disturbed self-awareness and depersonalization over and above the associations both constructs have with psychotic symptoms.

Our finding is in line with findings of two previous studies examining associations between insecure attachment and dissociation, a construct related to disturbed self-awareness and depersonalization, in a nonclinical sample (Laoide et al., 2018; Sandberg, 2010). They found significant positive associations between anxious attachment and dissociation, whereas avoidant attachment was not significantly associated (Laoide et al., 2018; Sandberg, 2010). Combined with these previous results, our findings suggest that especially anxious attachment is related to disturbed self-awareness and depersonalization.

### Interpretation of Our Findings and Proposed Mechanisms

The cross-sectional design of the study precludes the ability to make inferences regarding the direction of the relationship between attachment style and disturbed self-awareness and depersonalization. Given the developmental history of these constructs, it is likely that attachment style and disturbed self-awareness or depersonalization affect each other. Other factors such as negative schemas, mentalization, and social cognition may also play a part in the interrelation. Previous studies reported on the impact of insecure attachment on the development of negative schemas (Korver-Nieberg et al., 2013; Murphy et al., 2018). Anxious attachment is assumed to entail a profound negative schema of the self, whereas avoidant attachment is assumed to entail a profound negative schema of others (Korver-Nieberg et al., 2013). It is suggested that individuals with negative self-schemas are more likely to focus on their own vulnerability, whereas individuals with negative other-schemas are more likely to interpret interactions with others negatively and focus on maliciousness of others (Freeman and Garety, 2014; Murphy et al., 2018). One could speculate that a negative view of the self coexists with a more fragile sense of identity, which hampers identity when the self is challenged. There is also something to be said for surmising that a negative view of others makes individuals more vigilant to interpreting environmental cues as hostile, which leads to suspiciousness of the intention of others and may increase the liability of disturbances in self-awareness or depersonalization. Moreover, previous studies have found associations between insecure attachment and poorer mentalization (Pos et al., 2015; Varela et al., 2021) and social cognition (Sood et al., 2022) skills. It is postulated that secure attachment is fundamental in developing a coherent (narrative) self in order to be able to set clear boundaries between self and others (Luyten et al., 2020). As described previously, blurring of boundaries between self and others is part of disturbed self-awareness and depersonalization (Park and Baxter, 2022).

We can only speculate about why anxious attachment is more strongly associated with disturbed self-awareness and depersonalization than avoidant attachment in our study. One possible explanation is that higher levels of anxious attachment are more strongly linked to high arousal and excessive expression and at the same time insufficient ability to cope with it. This, in combination with the typically negative view of self, may increase the vulnerability of altered self-experiences. Future research may clarify whether stressful experiences or life events have a mediating impact on the association between attachment style and altered self-experiences. Another explanation might be that individuals with an avoidant attachment interpreted certain items on the questionnaires as a sign of weakness and therefore did not self-report this information. Perhaps they reported that they do not have altered self-experiences in order to maintain a positive self-view. It is also possible that the PAM items did not appear to adequately measure avoidant attachment, as was suggested by Olbert et al. (2016). However, we are cautious to assume this, as findings by Olbert et al. (2016) are not yet replicated in other samples and other studies did find good reliability for both subscales of the original PAM (Berry et al., 2006; Berry et al., 2008; Russo et al., 2018) and German version of the PAM (Kvrgic et al., 2012).

As mentioned, we assume that causality operates in the opposite direction as well. It seems self-evident that disturbances in self-awareness and the experience of depersonalization can hinder social interactions and the ability to attach to others. After all, an intact self-presence and boundary between self and others seem a prerequisite to engage in the social world (Nelson et al., 2009b). Or in other words, understanding mental states and intentions of others and being able to distinguish those from one's own mental states and intentions seem fundamental in being able to adequately relate to others (Park and Baxter, 2022). Moreover, there might be a self-reinforcing effect, disturbances in self-awareness, and depersonalization that may affect difficulties in relating to others, which in turn may undermine the sense of being grounded within a shared world (Nelson et al., 2009a) and may reinforce disturbances in self-awareness and depersonalization.

### Strengths and Limitations

In interpreting our findings, some limitations need to be taken into consideration. First, the naturalistic and cross-sectional design of the study does not allow for any conclusions regarding causation. Second, a more fundamental limitation is that the SELF is a self-report questionnaire and contains preformed questions with a restriction in answer possibilities (Likert scale) (Heering et al., 2016). We acknowledge that self-report instruments lack the clinical appreciation of interview-based assessment. Replication studies are needed to confirm our findings by using a more sophisticated and professional-based instrument as the EASE (Henriksen et al., 2021). However, the EASE is a time-consuming instrument that requires several days of training (Parnas et al., 2005), making the EASE less feasible in a routine clinical setting. Consequently, self-experiences are only assessed in some specialized clinics and disregarded in routine psychopathological examination. One could argue that assessing altered self-experience is so demanding that it never will gain momentum in diagnostics. However, evaluating self-report of self-experiences may make it possible to study these important phenomena in regular care and in larger cohort studies. We thus believe that assessing self-reported self-experiences with the SELF can be helpful as a first step. Similarly, a self-report questionnaire has been used to assess attachment style, liable to social desirability bias and self-report bias (Olbert et al., 2016). Third, when examining altered self-experiences, a linguistic expression and explicit awareness is inevitable, whereas self-experiences contain an ineffability at their core because they arise from a basic or non–self-reflexive level of experience and are difficult to express in language (Gawęda et al., 2018; Henriksen et al., 2021). Nevertheless, although we cautiously suggest that replication with the use of an interview-based instrument (the EASE) is needed, current findings indicate an important association between self-reported self-experience and attachment. Fourth, in the present study, we included participants from the Amsterdam site of GROUP as these participants had complete data on our predictor and outcome measures. We, therefore, cannot fully exclude the possibility of selection bias. Moreover, analyses were done in the overall group as we did not find an indication for significant differences among patients, siblings, and controls (nonsignificant attachment style × group interaction). However, the group of controls was smaller compared with the patient and sibling groups; therefore, we might have missed smaller differences, due to a lack of power. Future studies should replicate our findings in a larger representative sample of participants from different geographical areas and if possible conduct separate analyses per group with sufficient power. Lastly, in the current study, only the avoidant and anxious attachment dimensions of insecure attachment were captured. Recently, the PAM has been revised in order to add the dimension disorganized attachment to the two traditional dimensions (Pollard et al., 2020). It is of interest in future studies to replicate our findings by using this revised scale as the dimension disorganized attachment might be more closely associated with vulnerability to psychosis (Pollard et al., 2020).

### Clinical and Research Implications

From a phenomenological perspective, paranoid appraisals such as delusions might be representations of an individual's attempt to make sense of the experience of disturbed self-awareness and depersonalization. Perhaps it is so deeply disrupting if the basic self is disturbed, that paranoid appraisals or delusions are the best way to cope with these altered self-experiences (Nelson et al., 2020). Current therapeutic interventions are often targeted on positive symptoms, while potentially underlying disturbed self-awareness, depersonalization, and insecure attachment remain. Therefore, future studies on developing evidence-based interventions to improve self-awareness and depersonalization and attachment are needed. For now, clinicians may consider to focus on strengthening the interaction with the outside world to counteract the hyperreflexive self-focus, such as sports, work, and social contacts (Henriksen et al., 2021). Moreover, interventions focussed on strengthening the therapeutic alliance, and thereby increasing secure attachment representations might be a necessary step (Taylor et al., 2015). Possibly, shared exploring and verbalizing patients' experiences may contribute to a shared understanding of the association among anomalous self-experiences, attachment, and clinical features. This may generate understanding and improve therapeutic alliance.

Longitudinal studies in larger samples are required to clarify the direction of pathways identified in this study and address causality. Future studies could provide useful insight into the role of other psychological variables such as self and other schemas, mentalization, and social cognition within this relationship and across the psychosis continuum. Moreover, it might be interesting to investigate the impact of other confounders such as antipsychotic medication and cannabis use. In the present study, we examined associations in the total sample of participants since the group status (patient, sibling or control) × attachment interaction effects were not significant. This suggests a general association, irrespective of illness or vulnerability status and possibly not specific for patients with psychotic disorders. It is of interest to replicate these findings and examine the possible impact of illness duration and use of antipsychotic medication. Moreover, investigating the possibly differential impact of anxious and avoidant attachment on self-reported disturbed self-awareness and depersonalization outcome measures is an interesting topic for further research.

## CONCLUSIONS

In conclusion, we showed that attachment style is associated with self-reported disturbed self-awareness and depersonalization over and above the influence of psychotic and depressive experiences in people across the vulnerability spectrum of psychosis. Although we cannot address causality, the association between attachment style and disturbed self-awareness and depersonalization further supports the importance of attachment style in prevention and treatment interventions of patients with psychotic disorders or those with an increased vulnerability. Further longitudinal research investigating causal relationships is needed.

## Supplementary Material
