# Supplementary material for: Attachment Style and Self-Experience: The Association Between Attachment Style and Self-Reported Altered Self-Experience in Patients With Psychotic Disorders, Unaffected Siblings, and Healthy Controls
Source: J Nerv Ment Dis. 2023 Mar 24;211(6):440–7. doi: 10.1097/NMD.0000000000001634 (PMC10227941; doi:10.1097/NMD.0000000000001634)
Supplement: Supplementary file 1 [file jnmd-211-440-s001.docx]

**The association between attachment style and self-reported altered self-experience in patients with psychotic disorders, unaffected siblings and healthy controls.**

**Abstract**

The present study aimed to examine the cross-sectional association between attachment style and self-reported disturbed self-awareness (disturbed sense of mineness of experiences) and depersonalization (disturbed sense of first-person perspective) in patients with psychotic disorders, unaffected siblings, and healthy controls. Data pertains to a subsample of the Genetic Risk and Outcome of Psychosis (GROUP) Study. We found positive associations between anxious attachment and disturbed self-awareness and depersonalization across participants with different psychosis vulnerability. We also found a positive association between avoidant attachment and depersonalization, although on a trend level. Findings indicate that attachment style is associated with self-reported disturbed self-awareness and depersonalization over and above the influence of psychotic or depressive experiences in people across the vulnerability spectrum of psychosis. This supports the importance of attachment style, self-awareness and depersonalization as potential targets in prevention and treatment interventions in patients with psychotic disorders or those with increased vulnerability.

*Keywords: disturbed self-awareness, depersonalization; schizophrenia spectrum disorders.*

**Introduction**

Altered self-experiences are seen as a fundamental feature of schizophrenia spectrum disorders (Sass et al., 2013; Sass & Parnas, 2003) and may underlie several symptom dimensions such as positive, negative and disorganized psychotic symptoms (Parnas et al., 2005). Altered self-experiences capture a disturbed sense of self-presence and first-person perspective, disturbed self-awareness and disturbed common sense, among other experiences (Sass et al., 2013; Sass & Parnas, 2003). Several studies, including a recent systematic review (Henriksen et al., 2021) and three meta-analyses (Burgin et al., 2022; Hur et al., 2014; Raballo et al., 2021) consistently show that altered self-experiences are more common in schizophrenia spectrum disorders (including schizophrenia, non-affective psychotic disorders, and schizotypal disorders) compared to other mental disorders (Henriksen et al., 2021; Raballo et al., 2021) or healthy controls (Burgin et al., 2022; Henriksen et al., 2021; Hur et al., 2014; Raballo et al., 2021).

In the present study, we are interested in sub-aspects of altered self-experiences and more specifically in disturbed self-awareness and depersonalization. Disturbed self-awareness refers to the implicit awareness of an individual of being the owner of his body, perceptions, thoughts and experiences (sense of ownership) as well as an individual experiencing himself as the source or initiator of his thoughts, intentions and actions (sense of agency) (Zandersen & Parnas, 2019). Disturbed self-awareness in schizophrenia spectrum disorders is reflected in a reduced sense of ownership and agency and is hypothesized to have three main aspects: diminished self-affection, hyper-reflexivity and disturbed grip or hold (Sass, 2014; Sass & Parnas, 2003). Diminished self-affection refers to a decline in the experienced sense of existing as a subject of awareness or a decline in sense of ownership or agency. Hyper-reflexivity refers to an intensified awareness of phenomena that are normally in the background of experience or considered as part of oneself. Both self-awareness disturbances may lead to a disturbed sense of grip or hold on the inside and outside world (Parnas et al., 2005). Depersonalization refers to a feeling of detachment from one’s self (Parnas et al., 2005). In previous studies, depersonalization is often considered in the light of dissociative phenomena (Berry et al., 2017; Laoide et al., 2018; Mertens et al., 2021; Sandberg, 2010; Sheinbaum et al., 2014; Sheinbaum et al., 2020; van Dam et al., 2014), however, in the current study we are interested in depersonalization in the form of a disturbed sense of self-presence and first-person perspective (Parnas et al., 2005).

Recent studies consider altered self-experiences as important factors in the development of psychotic disorders (Cicero et al., 2017; Gawęda et al., 2018), hence knowledge concerning possible aetiological factors associated with altered self-experiences is important. There is a strong rationale to hypothesize an interrelation between attachment style and altered self-experiences. Experiences in (early) social interactions may affect how we see ourselves, others, and the world. The deeply ingrained beliefs or perceptions individuals hold about themselves and others are embedded in attachment theory (Bowlby, 1969). Attachment theory proposes that the pattern of interaction between infants and their primary caregivers leads to the development of mental representations about self and others (Ainsworth, 1978). These representations function as templates for thoughts, feelings, and behaviours in interactions with others throughout the lifespan (Brennan et al., 1998). Securely attached individuals have a secure foundation and believe that attachment figures are available when needed. They possess the ability to establish stable and long-term intimate relationships (Brennan et al., 1998). An insecure attachment style has been conceptualized in two underlying dimensions, anxious attachment and avoidant attachment (Brennan, 1998). Anxious attachment is characterized by a continuous desire for proximity and fear of rejection, making physical or emotional distance difficult to tolerate. Avoidant attachment is characterized by a continuous desire for independence and a fear of proximity, making physical or emotional closeness difficult to tolerate (Brennan et al., 1998). Previously Gaweda et al. (Gawęda et al., 2018) found both insecure attachment and self-disturbances to play a mediating role in the association between trauma and development of psychotic symptoms in a non-clinical population. The authors also reported a significant interrelation between anxious attachment and self-disturbance (Gawęda et al., 2018). Moreover, previous studies found positive associations between insecure attachment and dissociative phenomena (Laoide et al., 2018; Sandberg, 2010).

However, we are not aware of a study that explicitly investigated the association between attachment style and self-reported self-awareness or depersonalization in patients with psychotic disorders. Hence, in the present study, we aimed to examine this association in people with different vulnerability for psychosis, i.e. in a combined sample of patients with a non-affective psychotic disorder, unaffected siblings, and healthy controls. We hypothesized that a more insecure attachment style is associated with higher levels of disturbed self-awareness and depersonalization, and accounts for unique variance in these disturbances over and above the influence of psychotic and depressive experiences.

Ultimately, enhancing our understanding of factors associated with disturbed self-awareness or depersonalization may be informative for prevention and treatment interventions for persons with, or at risk of, psychotic disorders. Being aware of insecure attachment and its possible relation to the development of altered self-experiences may enlarge our empathic understanding of the experiential world of patients. This might strengthen the focus on the therapist-patient relationship and improve care with a positive effect on the development of a coherent and stable sense of self and a reduction of altered self-experiences.

**Methods**

*Study design and participants*
Patients included in the current study were part of the Genetic Risk and Outcome of Psychosis (GROUP) study. GROUP is a Dutch multi-site naturalistic follow-up study (after three and six years) designed to study risk and protective factors influencing the onset and course of psychotic disorders in patients, their unaffected family members, and non-related controls (Korver et al., 2012). Patients were recruited in selected representative geographical areas of the Netherlands and (the Dutch-speaking part of) Belgium. Random mailings to addresses were used to recruit controls in the selected areas. Interviewers received extensive training to optimize the reliability of measurements (Korver et al., 2012). Inclusion criteria were an age range from 16 to 50 years and mastery of the Dutch language. Patients had to meet the DSM-IV-TR criteria for a non-affective psychotic disorder, assessed with the Comprehensive Assessment of Symptoms and History (CASH) (Andreasen et al., 1992) or the Schedules for Clinical Assessment in Neuropsychiatry (SCAN) (Schutzwohl et al., 2007). The assessments were conducted by trained researchers. Participants were either investigated on site or (if necessary) assessments were conducted at their home. The same protocol was being used during in-home assessments and assessments on site. Average assessment time was four hours for patients and three hours for siblings and controls. The self-reported questionnaires (including the PAM and SELF) were sent to the participants prior to the assessment meeting in order for them to fill out and bring the questionnaires to the meeting. At the start of the assessment, the self-report questionnaires were checked for missing data. The full procedure of the study has been described elsewhere (Korver et al., 2012). The current add-on study has a cross-sectional design, using data from the second follow-up assessment (after six years). Participants from the Amsterdam site of GROUP participated in this study concerning attachment style, disturbed self-awareness and depersonalization. We included 74 patients, 78 siblings and 32 controls, thus the combined sample was 184 participants. Sociodemographic characteristics at baseline are presented in table 1. The sample consisted of 112 males and 70 females with a mean age of 35.6 (ranging from 22 – 61). Most participants identified as White, whereas others were Moroccan, Surinamese, Turkish, of another ethnicity, or mixed.

*Measurements*

*Disturbed self-awareness and depersonalization*
The Self-Experience Lifetime Frequency Scale (SELF) was used to assess self-reported self-experience phenomena (Heering et al., 2016) (see Supplementary Table S1, Supplemental Digital Content 1, http://links.lww.com/JNMD/A159). The SELF captures two aspects of altered self-experience, namely disturbed self-awareness and depersonalization. Eight items represent the domain disturbed self-awareness and six items represent the domain depersonalization. The SELF domain disturbed self-awareness contains items based on the Examination of Anomalous Self-Experience (EASE) (Henriksen et al., 2021) and the Comprehensive Assessment of At-Risk Mental States (CAARMS) (Yung et al., 2005). The SELF domain depersonalization contains items from the Depersonalization Severity Scale (DSS) (Simeon et al., 2001). In the EASE comparable items are captured under items 2.2 ‘distorted first-person perspective’ and 2.3 ‘other states of depersonalization’(Henriksen et al., 2021). Patients were asked to report about the life-time frequency and level of distress of the 12 items on a 5-point Likert rating scale. Frequency scores range from 0 (never) to 4 (continuously) and level of distress scores from 1 (no distress) to 5 (extremely distressed). A total score of the two subscales was calculated by adding the scores of both frequency and distress for each item, with higher scores reflecting higher levels of disturbed self-awareness and depersonalization. Good internal consistency of the two components has previously been demonstrated in a combined clinical and non-clinical sample, with Cronbach’s alpha of the two SELF domains ranging from 0.79 to 0.88 (Heering et al., 2016).

*Attachment style*
Attachment style was measured with the Dutch version of the Psychosis Attachment Measure (PAM) (Korver, 2014). The Dutch PAM is a 15-item self-report questionnaire with seven items reflecting avoidant attachment and eight reflecting anxious attachment. Items are rated on a 4-point Likert rating scale ranging from 0 (not at all) to 3 (very much) (Korver, 2014). Average item scores were calculated for avoidant and anxious attachment, with higher scores reflecting higher levels of insecure attachment (Berry, 2006; Korver, 2014). The Dutch PAM is observed to be an adequate measurement with high construct validity and reliability in a clinical and non-clinical sample. Chronbach’s alpha of the two PAM domains ranged from 0.70 to 0.83 (Korver, 2014).

*Covariates*
Sociodemographic data were evaluated using a self-reported questionnaire, specifically developed for the GROUP-study. To correct for possible confounding effects, covariates were selected a priori. Age and gender were added as covariates as well as positive symptoms, negative symptoms, and depressive symptoms, based on their putative association with attachment style or self-disturbance (Carr et al., 2018; Gumley et al., 2014; Haug et al., 2015; Pearse et al., 2020; Rasmussen et al., 2020; Værnes et al., 2021; van Dam et al., 2014). The frequency of psychotic experiences was measured with the Community Assessment of Psychotic Experiences (CAPE) (Konings et al., 2006). This self-report questionnaire assesses the frequency of psychotic and depressive symptoms over the past three years. A mean total score was calculated for the subscales positive symptoms, negative symptoms and depressive symptoms, with a higher score reflecting a higher frequency of symptoms. According to a meta-analysis, Cronbach’s alpha values of the three CAPE domains reported in previous studies had a meta-analytic mean ranging between 0.81 and 0.91 (Mark & Toulopoulou, 2016)

*Statistical analyses*
All analyses were performed using Statistical Package for the Social Sciences (IBM SPSS Statistics) version 28.0. A power analysis (desired probability level of 0.025 and statistical power level of 0.8, 6 predictors per model and an anticipated effect size of 0.15 ) was performed to estimate the necessary sample size. The anticipated effect size is determined based on previously found intercorrelations by Gaweda et al. (2018). The estimated minimum required sample size was 114. As our combined sample consisted of 184 participants, we assume our regression analyses have sufficient power to detect significant findings. A missing value analysis was conducted for item scores on the PAM and SELF (see Supplementary Table S2, Supplemental Digital Content 1, http://links.lww.com/JNMD/A159). To generate subscale scores for all participants, missing items were imputed with the logistic regression method using the Markov chain Monte Carlo algorithm. Number of imputations was set at five based on the low percentage of incomplete items. Outliers were not removed because we expected scores to vary greatly in the field, which should be reflected in our data. To check the assumptions of normality, linearity and homoscedasticity, we used probability plots and scatterplots of standardized residuals. Violations of normality were detected for both attachment style as well as disturbed self-awareness and depersonalization data, with positively skewed distributions, indicating clustering of scores at the lower ends. We compared the results of hierarchical linear multiple regression analyses after log transformation and square root transformation of the dependent and independent variables. Since the results of the untransformed and (log and square root) transformed models were similar, we chose to report only results of the models without transformation of the variables. Sample characteristics of participants were assessed using descriptive statistics. Correlations between attachment style and altered self-experience domains of the total sample and separate groups (patients, siblings and controls) were assessed using Pearson Correlation (Supplementary Table S3, Supplemental Digital Content 1, http://links.lww.com/JNMD/A159).

First, between-group differences in the association between attachment style and self-reported disturbed self-awareness and depersonalization were tested by using linear regression models. Therefore, attachment style x status group interaction effects were tested for the two outcome domains. Because these models did not show significant differences between status groups (see table 3), we subsequently fitted models investigating the association between attachment style and disturbed self-awareness and depersonalization for the combined sample of participants.
 Second, hierarchical linear multiple regression analyses were carried out to investigate the association between attachment style and disturbed self-awareness and depersonalization, with attachment styles as predictors and disturbed self-awareness and depersonalization as outcome measures. The a priori selected covariates were first added to the model in steps, examining whether they contributed to the model, while controlling for covariates in previous steps. In the first model age and gender were entered. In the second model, we entered the other covariates in the above-mentioned order before adding attachment style at the last step. All analyses were performed with separate models for anxious and avoidant attachment. Multi-collinearity was assessed by calculating the Variance Inflation Factor (VIF) for all models. To account for multiple testing, we used a Bonferroni correction to minimize the risk of type I errors. Therefore, the two-tailed significance threshold was set at 0.025 (0.05 divided by 2, given the two SELF outcome domains).

**Results**

*Clinical characteristics*Clinical characteristics regarding attachment style and self-reported disturbed self-awareness and depersonalization are presented in table 2. Correlations between attachment style and altered self-experience domains of the total sample and separate groups (patients, siblings and controls) are shown in Supplementary Table S3 (Supplemental Digital Content 1, http://links.lww.com/JNMD/A159). Our results indicate that anxious attachment was more strongly correlated to altered self-experiences than avoidant attachment across groups.

*Cross-sectional associations between attachment style and self-reported altered self-experiences*
Cross-sectional associations between attachment style and disturbed self-awareness and depersonalization in the total sample of participants were assessed by using hierarchical linear multiple regression models, while controlling for age, gender, positive symptoms, negative symptoms and depressive symptoms. The covariates were added to the model in steps, anxious and avoidant attachment were added in the last step. Only the final model is presented here (see table 4), for the detailed models with all subsequent steps see Supplementary Table S4 (Supplemental Digital Content 1, http://links.lww.com/JNMD/A159). We found that anxious attachment was positively associated with disturbed self-awareness (β = 4.048, p = 0.023) and depersonalization (β = 2.975, p = 0.011). Positive symptoms were positively associated with disturbed self-awareness (β = 11.899, p < 0.001) and depersonalization (β = 4.659, p = 0.004). The other covariates (age, gender, negative symptoms and depressive symptoms) were not significantly associated after applying Bonferroni correction. Avoidant attachment was positively associated with depersonalization on a trend level (β = 2.129, p = 0.051). Depersonalization was positively associated with positive symptoms (β = 4.745, p = 0.004). Avoidant attachment was not significantly associated with disturbed self-awareness, although avoidant attachment was positively associated with positive symptoms (β = 11.950, p < 0.001) and depressive symptoms (β = 5.775, p = 0.020).
 **Discussion**
*Summary of findings*The current study investigated the cross-sectional associations between attachment style and self-reported disturbed self-awareness and depersonalization in a sample of patients with a non-affective psychotic disorder, unaffected siblings and healthy controls. Our main finding is that attachment style is associated with self-reported disturbed self-awareness and depersonalization over and above the influence of psychotic and depressive experiences in people with different vulnerability for psychosis. More specifically, our results show that anxious attachment is associated with self-reported disturbed self-awareness and depersonalization and avoidant attachment is associated with self-reported depersonalization on a trend level.

*Our findings compared to findings of previous research*
To the best of our knowledge, this is the first study investigating the association between attachment style and self-reported disturbed self-awareness and depersonalization in people across the psychosis vulnerability spectrum. However, our results build on previous research by Gaweda et al. (2018), who reported a significant association between anxious attachment (measured with the PAM) and self-disorders (measured with the Inventory of Psychotic-like Anomalous Self-experiences) in a non-clinical population. The current study adds to the latter by including both a clinical and non-clinical population, while correcting for age, gender and positive, negative and depressive symptoms. Correcting for psychotic symptoms rather than using psychotic symptoms as outcome measure, allowed us to study associations between attachment style and disturbed self-awareness and depersonalization over and above the associations both constructs have with psychotic symptoms.
 Our finding is in line with findings of two previous studies examining associations between insecure attachment and dissociation, a construct related to disturbed self-awareness and depersonalization, in a non-clinical sample (Laoide et al., 2018; Sandberg, 2010). They found significant positive associations between anxious attachment and dissociation, whereas avoidant attachment was not significantly associated (Laoide et al., 2018; Sandberg, 2010). Combined with these previous results, our findings suggest that especially anxious attachment is related to disturbed self-awareness and depersonalization.

*Interpretation of our findings and proposed mechanisms*
The cross-sectional design of the study precludes the ability to make inferences regarding the direction of the relationship between attachment style and disturbed self-awareness and depersonalization. Given the developmental history of these constructs, it is likely that attachment style and disturbed self-awareness or depersonalization affect each other. Other factors such as negative schemas, mentalization and social cognition may also play a part in the interrelation. Previous studies reported on the impact of insecure attachment on the development of negative schemas (Korver-Nieberg et al., 2013; Murphy et al., 2018). Anxious attachment is assumed to entail a profound negative schema of the self while avoidant attachment is assumed to entail a profound negative schema of others (Korver-Nieberg et al., 2013). It is suggested that individuals with negative self-schemas are more likely to focus on their own vulnerability whereas individuals with negative other-schemas are more likely to interpret interactions with others negatively and focus on maliciousness of others (Freeman & Garety, 2014; Murphy et al., 2018). One could speculate that a negative view of the self coexists with a more fragile sense of identity, which hampers identity when the self is challenged. There is also something to be said for surmising that a negative view of others makes individuals more vigilant to interpreting environmental cues as hostile, which leads to suspiciousness of the intention of others and may increase the liability of disturbances in self-awareness or depersonalization. Moreover, previous studies have found associations between insecure attachment and poorer mentalization (Pos et al., 2015; Varela et al., 2021) and social cognition (Sood et al., 2022) skills. It is postulated that secure attachment is fundamental in developing a coherent (narrative) self in order to be able to set clear boundaries between self and others (Luyten et al., 2020). As described above, blurring of boundaries between self and others are part of disturbed self-awareness and depersonalization (Park & Baxter, 2022).

We can only speculate why anxious attachment is more strongly associated with disturbed self-awareness and depersonalization than avoidant attachment in our study. One possible explanation is that higher levels of anxious attachment are more strongly linked to high arousal and excessive expression and at the same time insufficient ability to cope with it. This, in combination with the typically negative view of self may increase the vulnerability of altered self-experiences. Future research may clarify whether stressful experiences or life events have a mediating impact on the association between attachment style and altered self-experiences. Another explanation might be that individuals with an avoidant attachment interpreted certain items on the questionnaires as a sign of weakness and therefore did not self-report this information. Perhaps they reported that they do not have altered self-experiences to maintain a positive self-view. It is also possible that the PAM items did not appear to adequately measure avoidant attachment, as was suggested by Olbert et al. (2016). However, we are cautious to assume this, as findings by Olbert et al. (2016) are not yet replicated in other samples and other studies did find good reliability for both subscales of the original PAM (Berry, 2006; Berry et al., 2008; Russo et al., 2018) and German version of the PAM (Kvrgic et al., 2012).

As mentioned, we assume that causality operates in the opposite direction as well. It seems self-evident that disturbances in self-awareness and the experience of depersonalization can hinder social interactions and the ability to attach to others. After all, an intact self-presence and boundary between self and others seem a prerequisite to engage in the social world (Nelson, Sass, Thompson, et al., 2009). Or in other words, understanding mental states and intentions of others and being able to distinguish those from one’s own mental states and intentions, seems fundamental in being able to adequately relate to others (Park & Baxter, 2022). Moreover, there might be a self-reinforcing effect, disturbances in self-awareness and depersonalization may affect difficulties in relating to others, which in turn may undermine the sense of being grounded within a shared world (Nelson, Sass, & Skodlar, 2009) and may reinforce disturbances in self-awareness and depersonalization.

*Strengths and limitations*
In interpreting our findings, some limitations need to be taken into consideration. First, the naturalistic and cross-sectional design of the study does not allow for any conclusions regarding causation. Second, a more fundamental limitation is that the SELF is a self-report questionnaire and contains pre-formed questions with a restriction in answer possibilities (Likert-scale) (Heering et al., 2016). We acknowledge that self-report instruments lack the clinical appreciation of interview-based assessment. Replication studies are needed to confirm our findings by using a more sophisticated and professional-based instrument as the Examination of Anomalous Self-Experience (EASE) (Henriksen et al., 2021). However, the EASE is a time-consuming instrument that requires several days of training (Parnas et al., 2005), making the EASE less feasible in a routine clinical setting. Consequently, self-experiences are only assessed in some specialised clinics and disregarded in routine psychopathological examination. One could argue that assessing altered self-experience is so demanding that it never will gain momentum in diagnostics. However, evaluating self-report of self-experiences may make it possible to study these important phenomena in regular care and in larger cohort studies. We thus believe that assessing self-reported self-experiences with the SELF can be helpful as a first step. Similarly, a self-report questionnaire has been used to assess attachment style, liable to social desirability bias and self-report bias (Olbert et al., 2016). Third, when examining altered self-experiences, a linguistic expression and explicit awareness is inevitable, while self-experiences contain an ineffability at their core because they arise from a basic or non-self-reflexive level of experience, and are difficult to express in language (Gawęda et al., 2018; Henriksen et al., 2021). Nevertheless, although we cautiously suggest that replication with the use of an interview-based instrument (the EASE) is needed, current findings indicate an important association between self-reported self-experience and attachment. Fourth, in the present study we included participants from the Amsterdam site of GROUP as these participants had complete data on our predictor and outcome measures. We, therefore, cannot fully exclude the possibility of selection bias. Moreover, analyses were done in the overall group as we did not find an indication for significant differences between patients, siblings and controls (non-significant attachment style x group interaction). However, the group of controls was smaller compared to the patient and sibling groups, therefore we might have missed smaller differences, due to a lack of power. Future studies should replicate our findings in a larger representative sample of participants from different geographical areas and if possible conduct separate analyses per group with sufficient power. Lastly, in the current study, only the avoidant and anxious attachment dimensions of insecure attachment were captured. Recently, the PAM has been revised in order to add the dimension disorganized attachment to the two traditional dimensions (Pollard et al., 2020). It is of interest in future studies to replicate our findings by using this revised scale as the dimension disorganized attachment might be more closely associated with vulnerability to psychosis (Pollard et al., 2020).

*Clinical and research implications*
From a phenomenological perspective, paranoid appraisals such as delusions might be representations of an individual’s attempt to make sense of the experience of disturbed self-awareness and depersonalization. Perhaps it is so deeply disrupting if the basic self is disturbed, that paranoid appraisals or delusions are the best way to cope with these altered self-experiences (Nelson et al., 2020). Current therapeutic interventions are often targeted on positive symptoms, while potentially underlying disturbed self-awareness, depersonalization and insecure attachment remain. Therefore, future studies on developing evidence-based interventions to improve self-awareness and depersonalization and attachment are needed. For now, clinicians may consider to focus on strengthening the interaction with the outside world to counteract the hyper-reflexive self-focus, such as sports, work and social contacts (Henriksen et al., 2021). Moreover, interventions focussed on strengthening the therapeutic alliance and thereby increasing secure attachment representations might be a necessary step (Taylor et al., 2015). Possibly, shared exploring, and verbalizing patients’ experiences may contribute to a shared understanding of the association between anomalous self-experiences, attachment and clinical features. This may generate understanding and improve therapeutic alliance.
 Longitudinal studies in larger samples are required to clarify the direction of pathways identified in this study and address causality. Future studies could provide useful insight into the role of other psychological variables such as self and other schemas, mentalization and social cognition within this relationship and across the psychosis continuum. Moreover, it might be interesting to investigate the impact of other confounders such as antipsychotic medication and cannabis use. In the present study we examined associations in the total sample of participants since the group status (patient, sibling or control) x attachment interaction effects were not significant. This suggests a general association, irrespective of illness or vulnerability status and possibly not specific for patients with psychotic disorders. It is of interest to replicate these findings and examine the possible impact of illness duration and use of antipsychotic medication. Moreover, investigating the possibly differential impact of anxious and avoidant attachment on self-reported disturbed self-awareness and depersonalization outcome measures is an interesting topic for further research.

**Conclusions**In conclusion, we showed that attachment style is associated with self-reported disturbed self-awareness and depersonalization over and above the influence of psychotic and depressive experiences in people across the vulnerability spectrum of psychosis. Although we cannot address causality, the association between attachment style and disturbed self-awareness and depersonalization further supports the importance of attachment style in prevention and treatment interventions of patients with psychotic disorders or those with an increased vulnerability. Further longitudinal research investigating causal relationships is needed.

**References**

Ainsworth, M. D. S., Blehar, M. C., Waters, E., & Wall, S. . (1978). Patterns of Attachment: A Psychological Study of the Strange Situation. *Hillsdale, Lawlence Erlbaum Associates*.

Andreasen, N. C., Flaum, M., & Arndt, S. (1992). The Comprehensive Assessment of Symptoms and History (CASH). An instrument for assessing diagnosis and psychopathology. *Arch Gen Psychiatry*, *49*(8), 615-623. <https://doi.org/10.1001/archpsyc.1992.01820080023004>

Berry, K. (2006). Attachment styles, interpersonal relationships and psychotic phenomena in a non-clinical student sample. In A. Wearden (Ed.), (Vol. 41, pp. 707-718). Personality and Individual Differences.

Berry, K., Barrowclough, C., & Wearden, A. (2008). Attachment theory: a framework for understanding symptoms and interpersonal relationships in psychosis. *Behav Res Ther*, *46*(12), 1275-1282. <https://doi.org/10.1016/j.brat.2008.08.009>

Berry, K., Varese, F., & Bucci, S. (2017). Cognitive Attachment Model of Voices: Evidence Base and Future Implications. *Front Psychiatry*, *8*, 111. <https://doi.org/10.3389/fpsyt.2017.00111>

Bowlby, J. (1969). Attachment and Loss: Volume I: Attachment, vol. 79. *The International Psycho-Analytical Library*, 1-401.

Brennan, K. A. (1998). Self report measurement of adult romantic attachment: an integrative overview. In C. L. Clark (Ed.): J.A.R.

Brennan, K. A., Clark, C. L., & Shaver, P. R. (1998). Self-report measurement of adult attachment: An integrative overview. *In J. A. Simpson & W. S. Rholes (Eds.), Attachment theory and close relationships (pp. 46-76). The Guilford Press.*

Burgin, S., Reniers, R., & Humpston, C. (2022). Prevalence and assessment of self-disorders in the schizophrenia spectrum: a systematic review and meta-analysis. *Scientific Reports*, *12*(1), 1165. <https://doi.org/10.1038/s41598-022-05232-9>

Carr, S. C., Hardy, A., & Fornells-Ambrojo, M. (2018). Relationship between attachment style and symptom severity across the psychosis spectrum: A meta-analysis. *Clin Psychol Rev*, *59*, 145-158. <https://doi.org/10.1016/j.cpr.2017.12.001>

Cicero, D. C., Neis, A. M., Klaunig, M. J., & Trask, C. L. (2017). The Inventory of Psychotic-Like Anomalous Self-Experiences (IPASE): Development and validation. *Psychol Assess*, *29*(1), 13-25. <https://doi.org/10.1037/pas0000304>

Freeman, D., & Garety, P. (2014). Advances in understanding and treating persecutory delusions: a review. *Soc Psychiatry Psychiatr Epidemiol*, *49*(8), 1179-1189. <https://doi.org/10.1007/s00127-014-0928-7>

Gawęda, Ł., Pionke, R., Krężołek, M., Prochwicz, K., Kłosowska, J., Frydecka, D., Misiak, B., Kotowicz, K., Samochowiec, A., Mak, M., Błądziński, P., Cechnicki, A., & Nelson, B. (2018). Self-disturbances, cognitive biases and insecure attachment as mechanisms of the relationship between traumatic life events and psychotic-like experiences in non-clinical adults - A path analysis. *Psychiatry Res*, *259*, 571-578. <https://doi.org/10.1016/j.psychres.2017.11.009>

Gumley, A. I., Taylor, H. E., Schwannauer, M., & MacBeth, A. (2014). A systematic review of attachment and psychosis: measurement, construct validity and outcomes. *Acta Psychiatr Scand*, *129*(4), 257-274. <https://doi.org/10.1111/acps.12172>

Haug, E., Øie, M., Andreassen, O. A., Bratlien, U., Nelson, B., Aas, M., Møller, P., & Melle, I. (2015). Anomalous self-experience and childhood trauma in first-episode schizophrenia. *Compr Psychiatry*, *56*, 35-41. <https://doi.org/10.1016/j.comppsych.2014.10.005>

Heering, H. D., Goedhart, S., Bruggeman, R., Cahn, W., de Haan, L., Kahn, R. S., Meijer, C. J., Myin-Germeys, I., van Os, J., & Wiersma, D. (2016). Disturbed Experience of Self: Psychometric Analysis of the Self-Experience Lifetime Frequency Scale (SELF). *Psychopathology*, *49*(2), 69-76. <https://doi.org/10.1159/000441952>

Henriksen, M. G., Raballo, A., & Nordgaard, J. (2021). Self-disorders and psychopathology: a systematic review. *Lancet Psychiatry*, *8*(11), 1001-1012. <https://doi.org/10.1016/s2215-0366(21)00097-3>

Hur, J. W., Kwon, J. S., Lee, T. Y., & Park, S. (2014). The crisis of minimal self-awareness in schizophrenia: a meta-analytic review. *Schizophr Res*, *152*(1), 58-64. <https://doi.org/10.1016/j.schres.2013.08.042>

Konings, M., Bak, M., Hanssen, M., van Os, J., & Krabbendam, L. (2006). Validity and reliability of the CAPE: a self-report instrument for the measurement of psychotic experiences in the general population. *Acta Psychiatr Scand*, *114*(1), 55-61. <https://doi.org/10.1111/j.1600-0447.2005.00741.x>

Korver-Nieberg, N., Fett, A. K., Meijer, C. J., Koeter, M. W., Shergill, S. S., de Haan, L., & Krabbendam, L. (2013). Theory of mind, insecure attachment and paranoia in adolescents with early psychosis and healthy controls. *Aust N Z J Psychiatry*, *47*(8), 737-745. <https://doi.org/10.1177/0004867413484370>

Korver, N. (2014). Adult attachment in samples of psychotic patients and siblings at high risk for psychosis: Validation of the ‘Psychosis Attachment Measure’. (L. d. Haan, Trans.). In: Attachment and psychosis [Doctoral thesis, Department of Psychiatry, University of Amsterdam].

Korver, N., Quee, P. J., Boos, H. B., Simons, C. J., & de Haan, L. (2012). Genetic Risk and Outcome of Psychosis (GROUP), a multi-site longitudinal cohort study focused on gene-environment interaction: objectives, sample characteristics, recruitment and assessment methods. *Int J Methods Psychiatr Res*, *21*(3), 205-221. <https://doi.org/10.1002/mpr.1352>

Kvrgic, S., Beck, E. M., Cavelti, M., Kossowsky, J., Stieglitz, R. D., & Vauth, R. (2012). Focusing on the adult attachment style in schizophrenia in community mental health centres: validation of the Psychosis Attachment Measure (PAM) in a German-speaking sample. *Int J Soc Psychiatry*, *58*(4), 362-373. <https://doi.org/10.1177/0020764011399004>

Laoide, A., Egan, J., & Osborn, K. (2018). What was once essential, may become detrimental: The mediating role of depersonalization in the relationship between childhood emotional maltreatment and psychological distress in adults. *Journal of Trauma & Dissociation*, *19*(5), 514-534. <https://doi.org/10.1080/15299732.2017.1402398>

Luyten, P., Campbell, C., Allison, E., & Fonagy, P. (2020). The Mentalizing Approach to Psychopathology: State of the Art and Future Directions. *Annual Review of Clinical Psychology*, *16*, 297-325. <https://doi.org/10.1146/annurev-clinpsy-071919-015355>

Mark, W., & Toulopoulou, T. (2016). Psychometric Properties of "Community Assessment of Psychic Experiences": Review and Meta-analyses. *Schizophr Bull*, *42*(1), 34-44. <https://doi.org/10.1093/schbul/sbv088>

Mertens, Y. L., Racioppi, A., Sheinbaum, T., Kwapil, T., & Barrantes-Vidal, N. (2021). Dissociation and insecure attachment as mediators of the relation between childhood emotional abuse and nonclinical paranoid traits. *Eur J Psychotraumatol*, *12*(1), 1888539. <https://doi.org/10.1080/20008198.2021.1888539>

Murphy, P., Bentall, R. P., Freeman, D., O'Rourke, S., & Hutton, P. (2018). The paranoia as defence model of persecutory delusions: a systematic review and meta-analysis. *Lancet Psychiatry*, *5*(11), 913-929. <https://doi.org/10.1016/s2215-0366(18)30339-0>

Nelson, B., Lavoie, S., Gawęda, Ł., Li, E., Sass, L. A., Koren, D., McGorry, P. D., Jack, B. N., Parnas, J., Polari, A., Allott, K., Hartmann, J. A., & Whitford, T. J. (2020). The neurophenomenology of early psychosis: An integrative empirical study. *Consciousness and Cognition*, *77*, 102845. <https://doi.org/10.1016/j.concog.2019.102845>

Nelson, B., Sass, L. A., & Skodlar, B. (2009). The phenomenological model of psychotic vulnerability and its possible implications for psychological interventions in the ultra-high risk ('prodromal') population. *Psychopathology*, *42*(5), 283-292. <https://doi.org/10.1159/000228837>

Nelson, B., Sass, L. A., Thompson, A., Yung, A. R., Francey, S. M., Amminger, G. P., & McGorry, P. D. (2009). Does disturbance of self underlie social cognition deficits in schizophrenia and other psychotic disorders? *Early Interv Psychiatry*, *3*(2), 83-93. <https://doi.org/10.1111/j.1751-7893.2009.00112.x>

Olbert, C. M., Penn, D. L., Reise, S. P., Horan, W. P., Kern, R. S., Lee, J., & Green, M. F. (2016). Assessment of attachment in psychosis: A psychometric cause for concern. *Psychiatry Res*, *246*, 77-83. <https://doi.org/10.1016/j.psychres.2016.09.020>

Park, S., & Baxter, T. (2022). Schizophrenia in the flesh: Revisiting schizophrenia as a disorder of the bodily self. *Schizophr Res*. <https://doi.org/10.1016/j.schres.2021.12.031>

Parnas, J., Møller, P., Kircher, T., Thalbitzer, J., Jansson, L., Handest, P., & Zahavi, D. (2005). EASE: Examination of Anomalous Self-Experience. *Psychopathology*, *38*(5), 236-258. <https://doi.org/10.1159/000088441>

Pearse, E., Bucci, S., Raphael, J., & Berry, K. (2020). The relationship between attachment and functioning for people with serious mental illness: a systematic review. *Nord J Psychiatry*, *74*(8), 545-557. <https://doi.org/10.1080/08039488.2020.1767687>

Pollard, C., Bucci, S., MacBeth, A., & Berry, K. (2020). The revised Psychosis Attachment Measure: Measuring disorganized attachment. *Br J Clin Psychol*, *59*(3), 335-353. <https://doi.org/10.1111/bjc.12249>

Pos, K., Bartels-Velthuis, A. A., Simons, C. J., Korver-Nieberg, N., Meijer, C. J., & de Haan, L. (2015). Theory of Mind and attachment styles in people with psychotic disorders, their siblings, and controls. *Aust N Z J Psychiatry*, *49*(2), 171-180. <https://doi.org/10.1177/0004867414546386>

Raballo, A., Poletti, M., Preti, A., & Parnas, J. (2021). The Self in the Spectrum: A Meta-analysis of the Evidence Linking Basic Self-Disorders and Schizophrenia. *Schizophr Bull*, *47*(4), 1007-1017. <https://doi.org/10.1093/schbul/sbaa201>

Rasmussen, A. R., Reich, D., Lavoie, S., Li, E., Hartmann, J. A., McHugh, M., Whitford, T. J., & Nelson, B. (2020). The relation of basic self-disturbance to self-harm, eating disorder symptomatology and other clinical features: Exploration in an early psychosis sample. *Early Interv Psychiatry*, *14*(3), 275-282. <https://doi.org/10.1111/eip.12850>

Russo, D. A., Stochl, J., Hodgekins, J., Iglesias-González, M., Chipps, P., Painter, M., Jones, P. B., & Perez, J. (2018). Attachment styles and clinical correlates in people at ultra high risk for psychosis. *British Journal of Psychology*, *109*(1), 45-62. <https://doi.org/10.1111/bjop.12249>

Sandberg, D. A. (2010). Adult attachment as a predictor of posttraumatic stress and dissociation. *Journal of Trauma & Dissociation*, *11*(3), 293-307. <https://doi.org/10.1080/15299731003780937>

Sass, L., Pienkos, E., Nelson, B., & Medford, N. (2013). Anomalous self-experience in depersonalization and schizophrenia: a comparative investigation. *Consciousness and Cognition*, *22*(2), 430-441. <https://doi.org/10.1016/j.concog.2013.01.009>

Sass, L. A. (2014). Self-disturbance and schizophrenia: structure, specificity, pathogenesis (Current issues, New directions). *Schizophr Res*, *152*(1), 5-11. <https://doi.org/10.1016/j.schres.2013.05.017>

Sass, L. A., & Parnas, J. (2003). Schizophrenia, consciousness, and the self. *Schizophr Bull*, *29*(3), 427-444. <https://doi.org/10.1093/oxfordjournals.schbul.a007017>

Schutzwohl, M., Kallert, T., & Jurjanz, L. (2007). Using the Schedules for Clinical Assessment in Neuropsychiatry (SCAN 2.1) as a diagnostic interview providing dimensional measures: cross-national findings on the psychometric properties of psychopathology scales. *Eur Psychiatry*, *22*(4), 229-238. <https://doi.org/10.1016/j.eurpsy.2006.10.005>

Sheinbaum, T., Kwapil, T. R., & Barrantes-Vidal, N. (2014). Fearful attachment mediates the association of childhood trauma with schizotypy and psychotic-like experiences. *Psychiatry Res*, *220*(1-2), 691-693. <https://doi.org/10.1016/j.psychres.2014.07.030>

Sheinbaum, T., Racioppi, A., Kwapil, T. R., & Barrantes-Vidal, N. (2020). Attachment as a mechanism between childhood maltreatment and subclinical psychotic phenomena: Results from an eight-year follow-up study. *Schizophr Res*, *220*, 261-264. <https://doi.org/10.1016/j.schres.2020.03.023>

Simeon, D., Guralnik, O., & Schmeidler, J. (2001). Development of a depersonalization severity scale. *Journal of Traumatic Stress*, *14*(2), 341-349. <https://doi.org/10.1023/a:1011169019614>

Sood, M., Carnelley, K. B., & Newman-Taylor, K. (2022). How does insecure attachment lead to paranoia? A systematic critical review of cognitive, affective, and behavioural mechanisms. *Br J Clin Psychol*. <https://doi.org/10.1111/bjc.12361>

Taylor, P., Rietzschel, J., Danquah, A., & Berry, K. (2015). Changes in attachment representations during psychological therapy. *Psychother Res*, *25*(2), 222-238. <https://doi.org/10.1080/10503307.2014.886791>

Værnes, T. G., Røssberg, J. I., Melle, I., Nelson, B., Romm, K. L., & Møller, P. (2021). Basic self-disturbance trajectories in clinical high risk for psychosis: a one-year follow-up study. *European Archives of Psychiatry and Clinical Neuroscience*. <https://doi.org/10.1007/s00406-021-01349-6>

van Dam, D. S., Korver-Nieberg, N., Velthorst, E., Meijer, C. J., & de Haan, L. (2014). Childhood maltreatment, adult attachment and psychotic symptomatology: a study in patients, siblings and controls. *Soc Psychiatry Psychiatr Epidemiol*, *49*(11), 1759-1767. <https://doi.org/10.1007/s00127-014-0894-0>

Varela, L. F., Wong, K. H. T., Shergill, S. S., & Fett, A. J. (2021). Attachment styles moderate Theory of Mind differences between persons with schizophrenia, first-degree relatives and controls. *Br J Clin Psychol*, *60*(3), 339-356. <https://doi.org/10.1111/bjc.12308>

Yung, A. R., Yuen, H. P., McGorry, P. D., Phillips, L. J., Kelly, D., Dell'Olio, M., Francey, S. M., Cosgrave, E. M., Killackey, E., Stanford, C., Godfrey, K., & Buckby, J. (2005). Mapping the onset of psychosis: the Comprehensive Assessment of At-Risk Mental States. *Aust N Z J Psychiatry*, *39*(11-12), 964-971. <https://doi.org/10.1080/j.1440-1614.2005.01714.x>

Zandersen, M., & Parnas, J. (2019). Identity Disturbance, Feelings of Emptiness, and the Boundaries of the Schizophrenia Spectrum. *Schizophr Bull*, *45*(1), 106-113. <https://doi.org/10.1093/schbul/sbx183>

**Table Titles**

Table 1. Baseline characteristics of the total sample and separate groups (patients, siblings and controls).

Table 2. Clinical characteristics regarding attachment style and self-reported disturbed self-awareness and depersonalization of the total sample and separate groups (patients, siblings and controls).

Table 3. Results of multiple linear regression models regarding the interaction between group status and attachment style and self-reported disturbed self-awareness and depersonalization.

Table 4. Results of hierarchical multiple linear regression models regarding the cross-sectional association between attachment style and self-reported disturbed self-awareness and depersonalization of the total sample of participants.
